# Impaired Sensitivity to Thyroid Hormones Is Associated With the Change of Abdominal Fat in Euthyroid Type 2 Diabetes Patients: A Retrospective Cohort Study

**DOI:** 10.1155/2024/8462987

**Published:** 2024-04-29

**Authors:** Bin Cao, Kun Li, Jing Ke, Dong Zhao

**Affiliations:** ^1^Center for Endocrine Metabolism and Immune Diseases, Beijing Luhe Hospital, Capital Medical University, Beijing 101149, China; ^2^Beijing Key Laboratory of Diabetes Research and Care, Beijing 101149, China

**Keywords:** body mass index, diabetes, subcutaneous fat area, thyroid hormone sensitivity, visceral fat area

## Abstract

**Background and Aims:** This study is aimed at investigating the potential correlation of thyroid hormone sensitivity with visceral fat area (VFA), subcutaneous fat area (SFA), and body mass index (BMI) among euthyroid type 2 diabetes mellitus (T2DM) subjects.

**Methods:** Thyroid hormone sensitivity indices were calculated by thyroid feedback quantile–based index (TFQI), TSH index (TSHI), thyrotropin thyroxine resistance index (TT4RI), and free thyroxine (fT4)/free triiodothyronine (fT3) ratio. These indices were then categorized into quartiles for analysis. The outcomes were the change rates in VFA, SFA, and BMI among the participants.

**Result:** The present study included 921 patients, with a median follow-up of 2.2 years. In multivariate linear regression, when compared to the first quartile, SFA demonstrated a notable decline in the fourth quartile of TFQI, TSHI, and TT4RI (*β* coefficient = −5.78, −7.83, and − 6.84 cm^2^ per year), while it significantly increased in the fourth quartile of fT4/fT3 ratio (*β* coefficient = 6.13 cm^2^ per year). Similarly, in the fourth quartile of TFQI, TSHI, and TT4RI, VFA decreased significantly, evidenced by *β* coefficients of −5.14, −4.80, and −4.08 cm^2^ per year. Yet, among the quartiles of the fT4/fT3 ratio, no discernible trend in VFA was observed. There was no significant association between indices of thyroid hormone sensitivity and change in BMI.

**Conclusion:** Impaired central sensitivity to thyroid hormones was significantly associated with the reduction of VFA and SFA, while impaired peripheral sensitivity was associated with an increase of SFA in euthyroid individuals with T2DM.

## 1. Introduction

The prevalence of type 2 diabetes mellitus (T2DM) has experienced an alarming increase on a global scale, resulting in significant financial burdens and adverse health outcomes [1]. The surge in T2DM incidence has been largely attributed to the escalating prevalence of obesity [2, 3]. Notably, body fat distribution has been strongly associated with T2DM and its associated complications [4–8].

Thyroid hormones, primarily free thyroxine (fT4) and free triiodothyronine (fT3), play a pivotal role in regulating metabolism [9, 10]. Serving as critical regulators, they target adipose tissues to improve serum lipid profiles and reduce fat accumulation [11]. Additionally, obesity also exerts influence on thyroid function. In individuals with obesity, elevated levels of thyroid stimulating hormone (TSH) and fT3 have been observed [12, 13].

Physiologically, the hypothalamic-pituitary-thyroid (HPT) axis operates via negative feedback mechanisms to maintain the dynamic stability of thyroid hormones [14, 15]. The manifestations of thyroid hormone resistance can be classified into central and peripheral phenomena. Central resistance affects the feedback loop set point in the central nervous system, while peripheral resistance reduces the metabolic effects of hormones [16]. In cases of thyroid hormone resistance, elevated levels of thyroid hormones coincide with increased TSH concentrations. Mild resistance to thyroid hormones has been recognized as a potential factor contributing to metabolic disorders in the euthyroid and subclinical hypothyroidism population [17, 18]. Four novel indices have been proposed to assess acquired resistance to thyroid hormones, including thyroid feedback quantile–based index (TFQI), thyrotroph thyroxine resistance index (TT4RI), TSH index (TSHI), and fT3/fT4 [16, 19].

Previous analyses have focused on the impact of thyroid hormones and TSH on obesity; limited research has investigated the association between thyroid hormone sensitivity and obesity indices, particularly in individuals with type 2 diabetes. In this study, our objective was to explore the relationship of thyroid hormone sensitivity with visceral fat area (VFA), subcutaneous fat area (SFA), and body mass index (BMI) in euthyroid patients with T2DM.

## 2. Method

### 2.1. Subjects and Study Design

This retrospective cohort study is aimed at investigating the association between thyroid hormone sensitivity and VFA, SFA, and BMI.

This retrospective study examined data from 921 T2DM patients enrolled at the Center for Endocrine Metabolism and Immune Diseases since October 2017. The patients were followed up approximately once a year, with a median follow-up of 2.2 years.

The inclusion criteria were as follows: (1) age between 18 and 75 years, (2) diagnosis of T2DM was based on standard criteria from the American Diabetes Association, (3) normal baseline TSH levels and thyroid function, and (4) availability of complete data on VFA, SFA, and BMI.

Patients were excluded from the study if they met any of the following criteria: (1) history of thyroid surgery, hyperthyroidism, subacute thyroiditis, Hashimoto's thyroiditis, or other disease which may result in abnormal thyroid function; (2) use of pharmaceuticals that could impact thyroid hormone concentration, such as antithyroid medications or corticosteroids; (3) presence of a malignant tumor or a life expectancy of less than 5 years; (4) experiencing acute complications of diabetes; (5) diagnosed with severe heart, kidney, or liver disease; and (6) pregnant or nursing women.

Informed consent was obtained from all participants in the study. The study was approved by the Institutional Review Board of Luhe Hospital, Capital Medical University.

### 2.2. Data Collection

Demographic characteristics, lifestyle factors, past medical history, and medication-related information were collected, including age, sex, duration of diabetes, smoking and drinking status, comorbidities, and medication details. Smoking status and drinking status were self-reported and categorized into two levels: current and never/former. Hypertension was defined as systolic blood pressure ≥ 140 mmHg or diastolic blood pressure ≥ 90 mmHg, or the use of any antihypertensive medications. BMI was calculated using the following formula: BMI = body weight in kilograms divided by the square of height in meters.

After an overnight fast of at least 8 hours, venous blood samples were collected for biochemical measurements. Serum levels of TSH, fT3, and fT4 were determined using a chemiluminescence assay performed on a Roche Cobas E601 analyzer. Glycosylated hemoglobin (HbA1c) was measured using a standardized high-performance liquid chromatography assay conducted on an ADAMS A1c HA-8180 analyzer. Serum creatinine (Cr) and low-density lipoprotein cholesterol (LDL-c) were measured using a chemiluminescence immunoassay performed on an AU5800 analyzer.

### 2.3. Measurement of SFA and VFA

VFA and SFA were assessed using dual bioelectrical impedance analysis (BIA) (HDS 2000; Omron Co. Ltd., Kyoto, Japan). Following an overnight fast of at least 8 hours, patients were positioned supine and asked to expose their ankles, wrists, and abdominal skin. Electrode detectors were then attached to the abdomen, wrist, and ankle. Patients were instructed to hold their breath for a few seconds, after which the VFA and SFA were measured. The measurement process adhered to standard protocols and was carefully supervised by a trained researcher. In case of any nonstandard procedure or the possibility of errors, an additional BIA measurement was conducted to ensure accuracy.

### 2.4. Definition of Change and Change Rate of VFA, SFA, and BMI

The change in BMI, SFA, and VFA was calculated by subtracting the baseline value from the value at the last visit. The change rate of BMI, SFA, and VFA was determined by dividing the change by the interval between the baseline and the last visit.

### 2.5. Indices of Thyroid Hormone Sensitivity

Normal range of TSH, fT3, and fT4 was 0.27–4.2 mIU/L, 3.1–6.8 pmol/L, and 12–22 pmol/L, respectively. Given the difficulty in directly measuring thyroid hormone sensitivity in clinical practice, we employed the TFQI, TSHI, TT4RI, and fT4/fT3 ratio as surrogate markers. These indices have been validated across numerous studies, underscoring their clinical value and accuracy in evaluating the sensitivity to thyroid hormone [20–22]. Indices of central thyroid hormone sensitivity included the TFQI (TFQI = cdf fT4 − (1 − cdf TSH), where cdf is the cumulative distribution function), TSHI (TSHI = ln TSH (mIU/L) + 0.1345 × fT4 (pmol/L)), and TT4RI (TT4RI = fT4 (pmol/L) × TSH (mIU/L)), and peripheral thyroid sensitivity was calculated as fT4/fT3 [19]. For TFQI, TSHI, TT4RI, and fT4/fT3 ratio, higher values indicated lower thyroid hormone sensitivity. The patients were categorized into four groups based on quartiles of thyroid parameters. Quartile ranges for these indices are as follows: The TFQI was divided into quartiles as follows: Q1 (≤ −0.962 to < −0.277), Q2 (≤ −0.277 to < −0.008), Q3 (≤ −0.008 to < 0.270), and Q4 (≤ 0.270 to < 0.972). Quartiles for the TSHI were established as follows: Q1 (≤ 0.761 to < 2.364), Q2 (≤ 2.364 to < 2.732), Q3 (≤ 2.732 to < 3.125), and Q4 (≤ 3.125 to < 4.229). The TT4RI quartiles were defined as follows: Q1 (≤ 4.587 to < 19.540), Q2 (≤ 19.540 to < 27.325), Q3 (≤ 27.325 to < 39.273), and Q4 (≤ 39.273 to < 84.652). Finally, the fT4/fT3 ratio was categorized as follows: Q1 (≤ 2.459 to < 3.264), Q2 (≤ 3.264 to < 3.620), Q3 (≤ 3.620 to < 4.015), and Q4 (≤ 4.015 to < 5.796).

### 2.6. Statistical Analysis

Continuous variables were presented as mean ± standard deviation (SD) or median (interquartile range (IQR)) values, and categorical variables were presented as frequency (%). *P* for trend was calculated by linear regression analyses. The independent association of thyroid hormone sensitivity and change rate of VFA and SFA was evaluated by multivariable linear regression models. We constructed the models with adjustments for major covariables: age, sex, duration of diabetes, HbA1c, antihyperglycemic agents, history of hypertension, smoking status, drinking status, and LDL-c. All analyses were performed using R software (version 4.2.1). A two-sided *P* value < 0.05 was considered statistically significant.

## 3. Results

### 3.1. General Characteristic of Study Population

As illustrated in Figure S1, a total of 1140 patients had available VFA and SFA measurements. Among them, 219 patients were excluded based on the predefined exclusion criteria, resulting in a final cohort of 921 patients enrolled between October 2017 and December 2021. The baseline characteristics of the study groups are presented in Table 1. The mean age of the population was 51.16 ± 11.92 years, with a median diabetes duration of 4.5 years, and 554 (60.2%) were men. The median follow-up duration was 2.2 years. At the last follow-up visit, the average change rate of VFA, SFA, and BMI was observed to be −0.40 cm^2^ per year, −4.52 cm^2^ per year, and 0.14 kg/m^2^ per year, respectively.

### 3.2. Trend Test for the Associations Between Thyroid Hormone Sensitivity and Changes in VFA, SFA, and BMI

**Figure 1:**
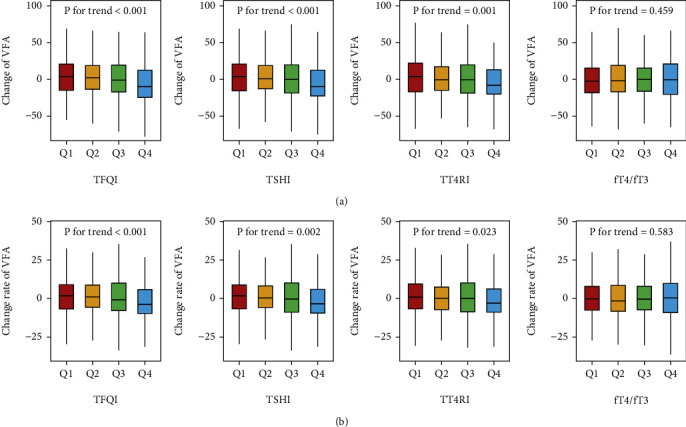
The distributions of the (a) absolute change and (b) change rate of VFA among the quartile groups according to thyroid hormone sensitivity.

Figure 1 displays the changes in VFA among the quartile groups based on thyroid hormone sensitivity indices. A significant decreasing trend in VFA was observed across the increasing quartiles of TFQI (3.61, 2.19, −1.07, and −9.83 cm^2^), TSHI (3.84, 0.78, −0.12, and −9.60 cm^2^), and TT4RI (3.85, −0.35, −0.43, and −8.18 cm^2^) (Figure 1(a)). The same trend was also observed in the change rate of VFA, with values of 1.75, 1.14, −0.82, and −3.71 cm^2^ per year for TFQI; 1.73, 0.34, −0.25, and −3.44 cm^2^ per year for TSHI; and 0.97, 0.30, 0.10, and −2.99 cm^2^ per year for TT4RI (Figure 1(b)).

**Figure 2:**
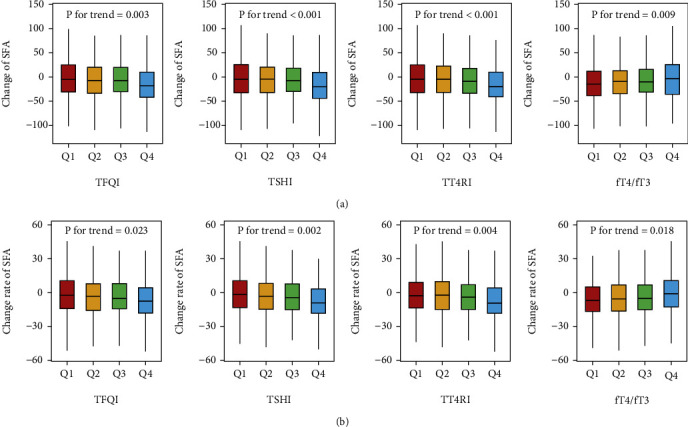
The distributions of the (a) absolute change and (b) change rate of SFA among the quartile groups according to thyroid hormone sensitivity.

Figure 2 illustrates the changes in SFA. The decline in SFA increased with higher quartiles of TFQI (−5.27, −7.18, −7.61, and −17.92 cm^2^), TSHI (−5.03, −4.34, −8.14, and −20.38 cm^2^), and TT4RI (−4.63, −4.49, −8.90, and −19.87 cm^2^). However, as the quartiles of the fT4/fT3 ratio increased, the decline in SFA became less pronounced (−14.92, −9.47, −10.04, and −3.48 cm^2^) (Figure 2(a)). In Figure 2(b), the decline rate of SFA also increased with higher quartiles of TFQI (−2.42, −3.06, −5.13, and −7.46 cm^2^ per year), TSHI (−1.67, −3.12, −4.33, and −8.96 cm^2^ per year), and TT4RI (−2.64, −2.16, −3.94, and −9.33 cm^2^ per year). Similarly, as the quartiles of the fT4/fT3 ratio increased, the decline rate in SFA became less pronounced (−6.72, −5.40, −5.03, and −0.91 cm^2^ per year). However, no significant trend was observed between thyroid hormone sensitivity indices and changes in BMI (Figure S2).

### 3.3. The Linear Associations Between the Thyroid Hormone Sensitivity Indices and the Change Rate of VFA and SFA

**Figure 3:**
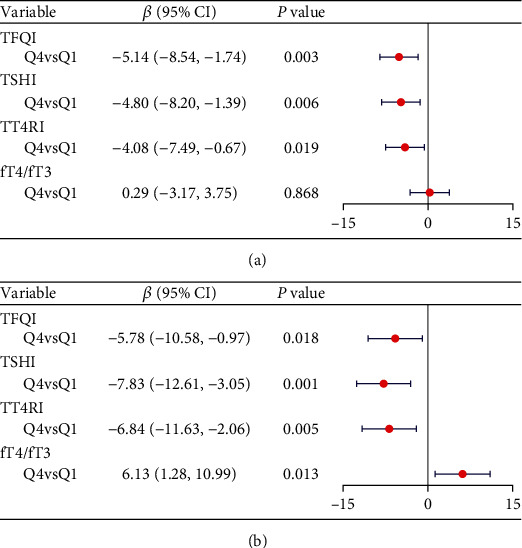
Multivariate linear regression of the association between thyroid hormone sensitivity indices and the decline rate of (a) VFA and (b) SFA. *β* coefficients were adjusted for age, sex, duration of diabetes, HbA1c, LDL-c, antihyperglycemic agents, history of hypertension, smoking status, and drinking status.

After adjusting for all confounders, compared to the first quartile, the fourth quartile showed a significant association with a decrease of −5.14 cm^2^ per year in the TFQI group, a decrease of −4.80 cm^2^ per year in the TSHI group, and a decrease of −4.08 cm^2^ per year in the TT4RI group. However, no significant association was observed between the change rate of VFA and the fT4/fT3 ratio (Figure 3(a)).

Similarly, in terms of SFA, compared to the first quantile, the fourth quantile showed a significant association with a decrease of −5.78 cm^2^ per year in the TFQI group, a decrease of −7.83 cm^2^ per year in the TSHI group, a decrease of −6.84 cm^2^ per year in the TT4RI group, and an increase of 6.13cm^2^ per year in the fT4/fT3 ratio (Figure 3(b)).

### 3.4. Subgroup Analysis Based on Gender

**Figure 4:**
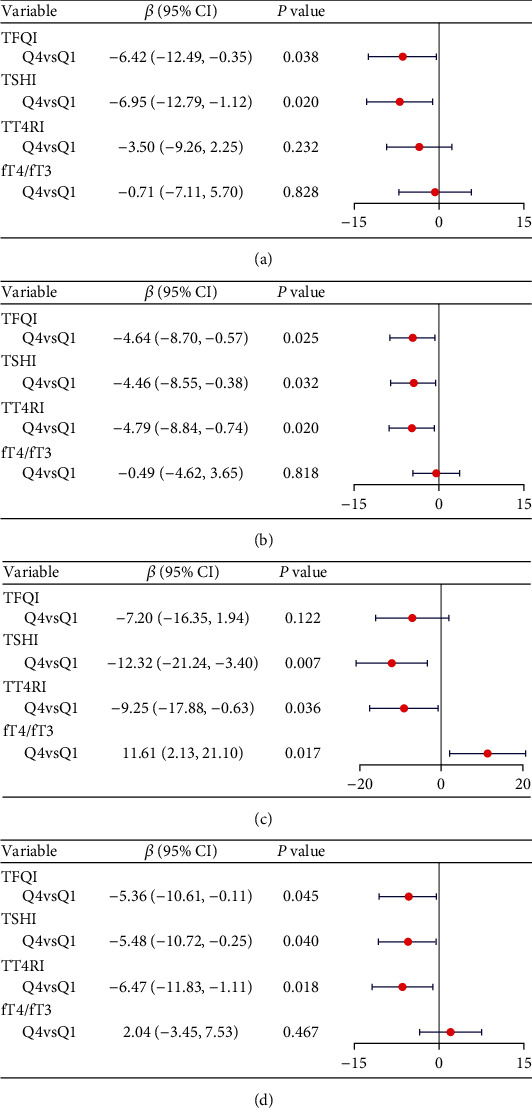
Multivariable linear regression of the association between thyroid hormone sensitivity indices and the change rate of VFA in females (a) and males (b), as well as the associations between thyroid hormone sensitivity indices and the change rate of SFA in females (c) and males (d). *β* coefficients were adjusted for age, sex, duration of diabetes, HbA1c, LDL-c, antihyperglycemic agents, history of hypertension, smoking status, drinking status.

Considering the influence of thyroid hormones on gender, subgroup analysis was conducted based on gender in this study. In both females (Figure 4(a)) and males (Figure 4(b)), the fourth quantile exhibited a significant association with a decline of VFA for TFQI and TSHI compared to the first quantile. In males, the fourth quantile was also significantly associated with a decline of VFA for TT4RI. However, no significant association was observed between the change of VFA and the fT4/fT3 ratio.

As depicted in Figure 4(c), among females, the fourth quantile demonstrated a significant association with the change rate of SFA for TSHI, TT4RI, and fT4/fT3 ratio compared to the first quantile. In males (Figure 4(d)), the fourth quantile exhibited a significant association with the change rate of SFA for central thyroid hormone sensitivity indices, while the fT4/fT3 ratio showed no association with the change rate of SFA.

## 4. Discussion

To the best of our knowledge, this is the first cohort study that presents evidence regarding the association between thyroid hormone sensitivity and changes in body fat distribution among individuals with T2DM. Specifically, we found that greater impairment in central sensitivity to thyroid hormones was linked to an accelerated decline rate of VFA and SFA. Moreover, higher peripheral sensitivity to thyroid hormones was associated with an increased rate of SFA. Notably, these associations were observed within the normal ranges of fT3, fT4, and TSH. These findings shed new light on our understanding of the influence of thyroid hormones on changes in body fat.

Thyroid hormones and TSH levels are regulated by HPT axis [23]. Given that thyroid hormones play a role in regulating the resting metabolic rate, thermogenesis, and lipolysis [24–26], alterations in thyroid hormone levels may indicate an adaptive process in obesity. Furthermore, research has shown that even slight variations in TSH levels within the normal range of thyroid function are associated with visceral obesity and BMI [27–29]. Currently, thyroid function is primarily assessed by measuring serum levels of TSH and thyroid hormones. However, considering the sensitivity of thyroid hormones, rather than relying solely on the individual levels of thyroid hormones, can offer new perspectives on the regulation of thyroid hormone homeostasis.

Thyroid hormone sensitivity can be categorized into central sensitivity, which reflects the feedback loop within the central nervous system, and peripheral sensitivity, which pertains to the metabolic effects of thyroid hormones [19]. A cross-sectional analysis revealed a negative association between TSHI, representing central sensitivity, and overall obesity, rather than central adiposity [30]. These findings suggest that impaired sensitivity to thyroid hormones increases the risk of overall obesity in euthyroid individuals. Our findings, however, differ from the previous study. When comparing our results to theirs, several factors need to be considered. Firstly, the previous study utilized a cross-sectional design, while ours employed a retrospective cohort design. Secondly, the previous research included subjects with subclinical hypothyroidism, whereas our study focused on euthyroid individuals with T2DM. Moreover, other central thyroid hormone sensitivity indices, such as TFQI, and peripheral thyroid hormone resistance indices were not evaluated in the previous study. The variations in population characteristics, study design, and observed indicators may contribute to these differences.

The pathogenesis of obesity is attributed not solely to overall fat content but also to fat distribution. Visceral fat, specifically, has a strong association with insulin resistance [31, 32] and is widely recognized as a significant risk factor for the development of comorbidities associated with obesity [33–35]. As this study represents the first investigation into the longitudinal relationship between thyroid hormone sensitivity and abdominal fat content, there is limited availability of comparative data. Nie et al. reported an independent and positive association between SFA and higher peripheral thyroid hormone sensitivity [36]. However, our findings demonstrate a negative relationship between peripheral thyroid hormone sensitivity and SFA. In individuals with impaired central sensitivity to thyroid hormone, particularly in relation to TSH, the response to T4 is compromised. Consequently, a relatively higher level of fT4 is required to achieve the same TSH level. Additionally, impaired peripheral sensitivity to thyroid hormone is indicative of reduced conversion of T4 to T3. Notably, the biological activity of fT3 is greater than that of fT4. Therefore, it is reasonable to hypothesize that impaired central sensitivity to thyroid hormone is associated with a greater reduction in both VFA and SFA, while impaired peripheral sensitivity is linked to a greater increase in SFA.

The major strengths of our study include the longitudinal study design and the relatively comprehensive indicators for thyroid function. However, there are also certain limitations that should be acknowledged. Firstly, the study utilized a retrospective cohort design, which may introduce potential biases. Additionally, the impact of different types of hypoglycemic drugs on the outcomes was not accounted for, as changes in medication during the follow-up period were not considered in our analyses. Furthermore, while computed tomography (CT) is considered the gold standard for evaluating abdominal fat distribution, its use should be limited due to concerns regarding radiation exposure. In our study, we opted to use BIA to measure VFA and SFA due to its accessibility and safety. Besides, whether participants had Hashimoto's thyroiditis was obtained through standardized questionnaires, yet not all participants underwent testing for TPO and TG antibodies. Although the study exclusively involved individuals with normal thyroid function and excluded those with a history of Hashimoto's thyroiditis to minimize the disease's potential effects, the potential impact of Hashimoto's thyroiditis cannot be completely ruled out. Lastly, although we observed a relationship between thyroid hormone sensitivity and abdominal fat, the specific underlying mechanism for this phenomenon remains unclear.

## 5. Conclusion

In summary, our study reveals significant associations between impaired central sensitivity to thyroid hormones and a reduction in VFA and SFA among euthyroid individuals with T2DM. Furthermore, impaired peripheral sensitivity to thyroid hormones was associated with an increase in SFA. The scientific significance of this study lies in its potential to inspire innovative approaches for managing fat accumulation in patients with T2DM, offering a novel perspective on optimizing metabolic health.

## Figures and Tables

**Table 1:** Characteristics of patients at baseline.

	**Characteristics (** **n** = 921**)**
At baseline	
Age (years)	50.43 (11.86)
Duration (years)	4.5 (0.4, 10.9)
Male (*n*, %)	554 (60.2)
Current smoker (*n*, %)	313 (34.1)
Current drinker (*n*, %)	374 (40.7)
Hypertension (*n*, %)	366 (40.5)
BMI (kg/m^2^)	26.98 (4.03)
VFA (cm^2^)	108.21 (43.50)
SFA (cm^2^)	209.63 (71.18)
Laboratory test	
HbA1c	8.18 (2.69)
Cr (*μ*mol/L)	67.26 (15.70)
LDL-c (mmol/L)	3.03 (0.90)
fT4 (pmol/L)	16.73 (1.97)
fT3 (pmol/L)	4.61 (0.61)
TSH (mIU/L)	1.83 (0.88)
Antihyperglycemic agent	
Insulin/sulfonylureas (*n*, %)	693 (75.2)
GLP1-RA/SGLT2i (*n*, %)	34 (3.7)
At last visit of follow-up	
BMI (kg/m^2^)	26.92 (6.76)
VFA (cm^2^)	106.94 (41.59)
SFA (cm^2^)	200.15 (69.34)
Change of BMI (kg/m^2^)	−0.07 (5.79)
Change of VFA (cm^2^)	−1.27 (32.26)
Change of SFA (cm^2^)	−9.48 (43.85)
Change rate of BMI (kg/m^2^ per year)	0.14 (6.37)
Change rate of VFA (cm^2^ per year)	−0.40 (18.04)
Change rate of SFA (cm^2^ per year)	−4.52 (25.55)
Follow-up period (years)	2.2 (1.8, 3.0)

Abbreviations: BMI, body mass index; fT3, free triiodothyronine; fT4, free thyroxine; GLP1-RA, glucagon-like peptide-1 receptor agonists; LDL-c, low-density lipoprotein cholesterol; SFA, subcutaneous fat area; SGLT2i, sodium-glucose cotransporter 2 inhibitors; TSH, thyroid stimulating hormone; VFA, visceral fat area.

## Data Availability

The data used to support the findings of this study are available from the corresponding author upon request.

## Supporting Information

Figure S1 Flow chart of the study population.

Figure S2 The distributions of the absolute change (A) and change rate (B) of BMI among the quartile groups according to thyroid hormone sensitivity.
