# Supplementary material for: Impaired Sensitivity to Thyroid Hormones Is Associated With the Change of Abdominal Fat in Euthyroid Type 2 Diabetes Patients: A Retrospective Cohort Study
Source: J Diabetes Res. 2024 Apr 29;2024:8462987. doi: 10.1155/2024/8462987 (PMC11073852; doi:10.1155/2024/8462987)
Supplement: Figure S1 — Flow chart of the study population. [file 8462987.f1.pdf]

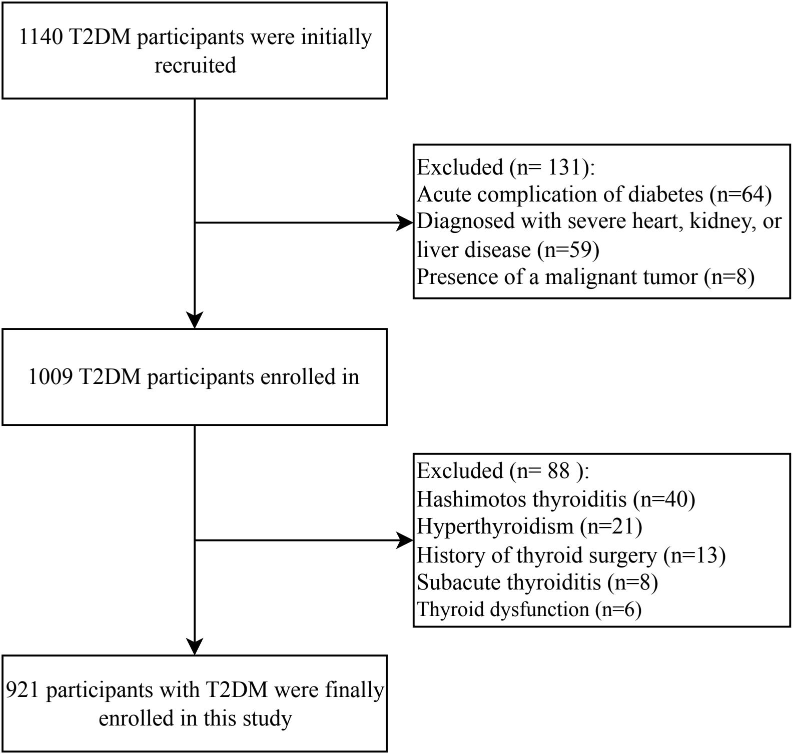
1140 T2DM participants were initially recruited
Excluded (n= 131):
Acute complication of diabetes (n=64)
Diagnosed with severe heart, kidney, or liver disease (n=59)
Presence of a malignant tumor (n=8)
1009 T2DM participants enrolled in
Excluded (n= 88):
Hashimotos thyroiditis (n=40)
Hyperthyroidism (n=21)
History of thyroid surgery (n=13)
Subacute thyroiditis (n=8)
Thyroid dysfunction (n=6)
921 participants with T2DM were finally enrolled in this study
