# Supplementary figures and images for: Impaired Sensitivity to Thyroid Hormones Is Associated With the Change of Abdominal Fat in Euthyroid Type 2 Diabetes Patients: A Retrospective Cohort Study
Source: J Diabetes Res. 2024 Apr 29;2024:8462987. doi: 10.1155/2024/8462987 (PMC11073852; doi:10.1155/2024/8462987)

A

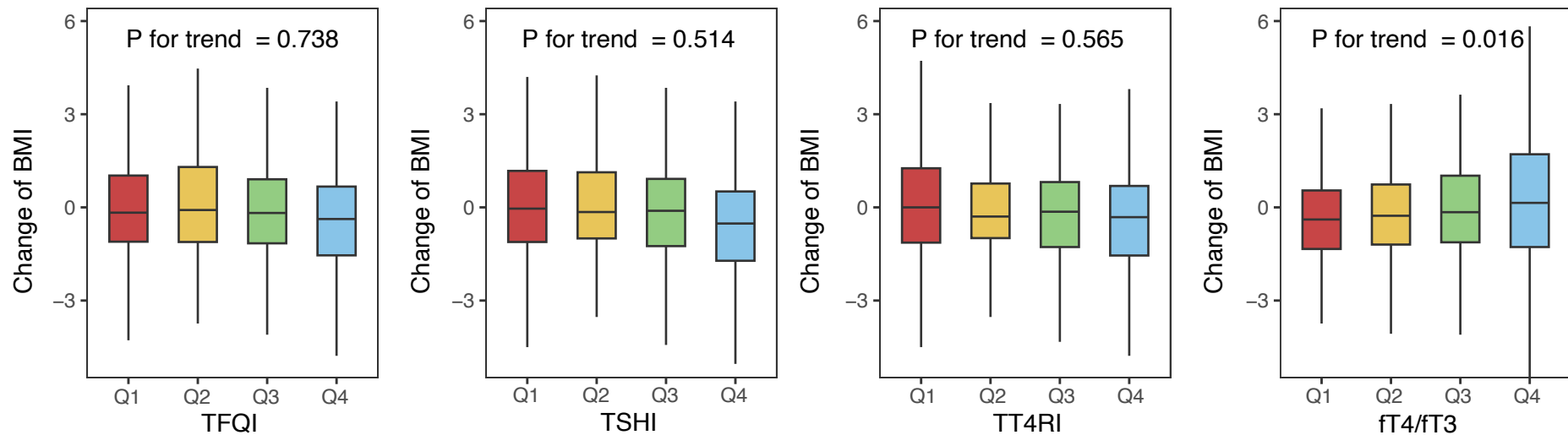

B

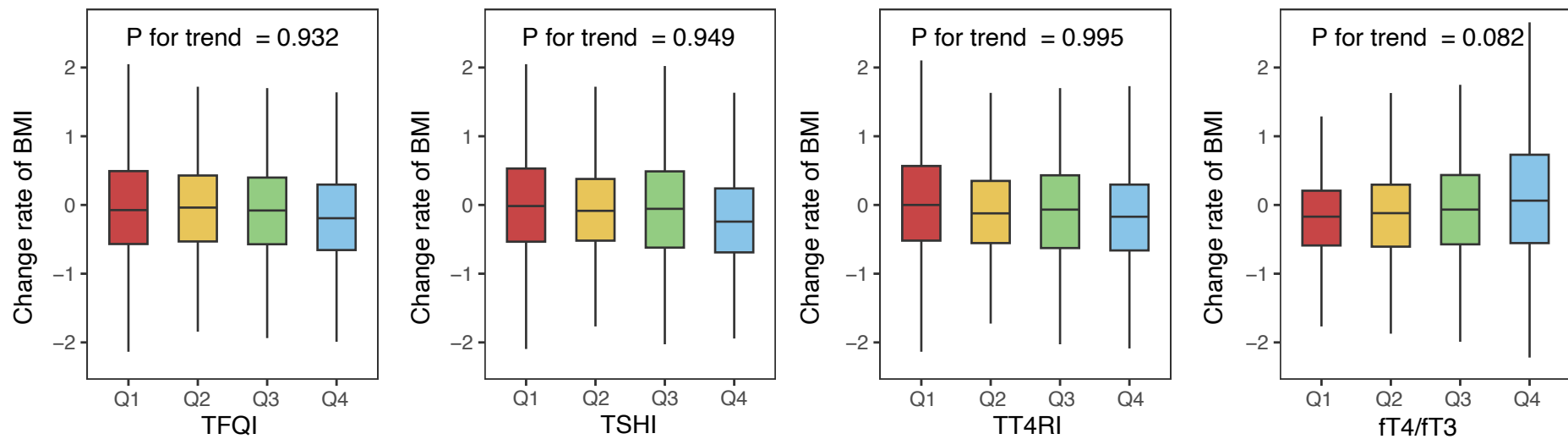

Supplement: Figure S2 — The distributions of the absolute change (A) and change rate (B) of BMI among the quartile groups according to thyroid hormone sensitivity. [file 8462987.f2.pdf]
